# Terry L. Erwin: She Had a Black Eye and in Her Arm She Held a Skunk: Re-published from "*American Entomologist*" Vol. 61 (I) on the occasion of ZooKeys 500

**DOI:** 10.3897/zookeys.500.9772

**Published:** 2015-04-27

**Authors:** Marlin E. Rice

**Affiliations:** 1 DuPont Pioneer, Johnston, Iowa, USA

Terry L. Erwin is Curator of Coleoptera at the Smithsonian Institution, National Museum of Natural History, in Washington, D.C. and editor-in-chief of *ZooKeys*. He generated significant controversy in 1982—which continues to this day—when he published an estimate of 30 million species on Earth, which was substantially more than the nearly one million described species. He was born 1 December 1940 in St. Helena, California and spent his youth trout fishing with his maternal grandfather in the High Sierra near Lake Tahoe. As a teenager, with prodding from his father, he built hot rod cars and was a founding member and later President of the California Conquistadores, a hot rod club in the San Francisco Bay area. Erwin earned his B.S. (1964, Biology) and M.A. (1966, Biology) degrees from San Jose State College. With a desire to learn from the three greatest living carabidologists, he first obtained a Ph.D. (1969, Entomology) from University of Alberta under the direction of George Ball. This was followed by a post-doctoral fellowship at Harvard University’s Museum of Comparative Zoology with Philip J. Darlington, Jr. During that year, a position opened at the (then) United States National Museum in the Department of Entomology, which he accepted, but two months after taking the job, he departed for a year-long sabbatical at Lund University in Sweden, where he completed the carabidology “trifecta” under the mentorship of Carl H. Lindroth. While in Sweden, the Chairman of Entomology at the USNM changed from Karl Krombien to Paul D. Hurd, who saw on his desk a proposal left by Terry to study California carabid beetles. Learning that grant money was available for research in Central America, Hurd crossed out “California” and wrote in “Panama.” Terry returned to Washington in 1971, as the second coleopterist within the USNM, and was greatly surprised to find that his proposal had been changed, funding had been secured, and he was scheduled for the next flight to the Canal Zone. Thus began a lifetime career on studies of insect biodiversity in neotropical forests.

**Figure:**
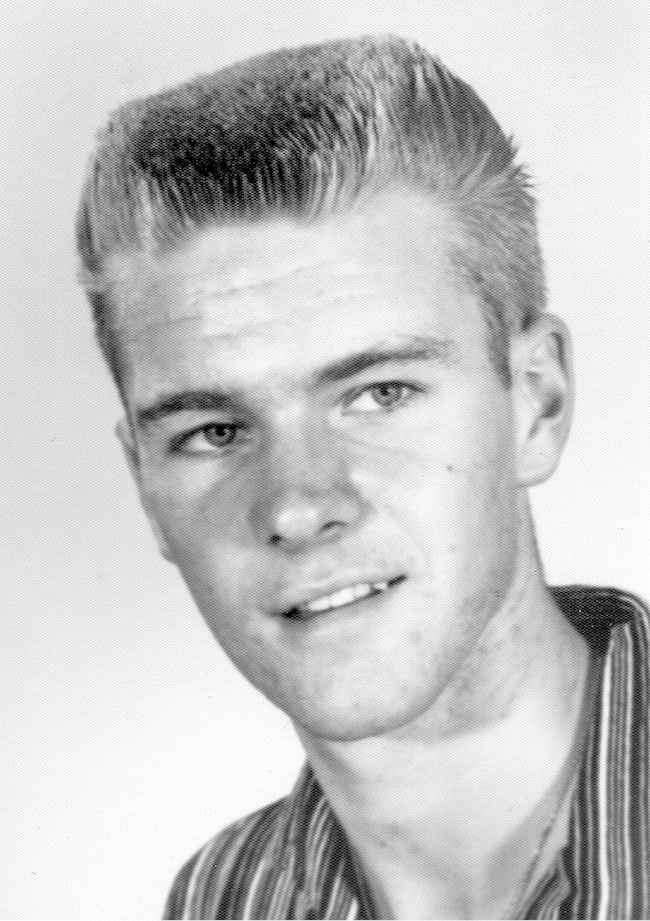
Terry Erwin, senior, Vallejo Senior High School, 1958.

**Figure:**
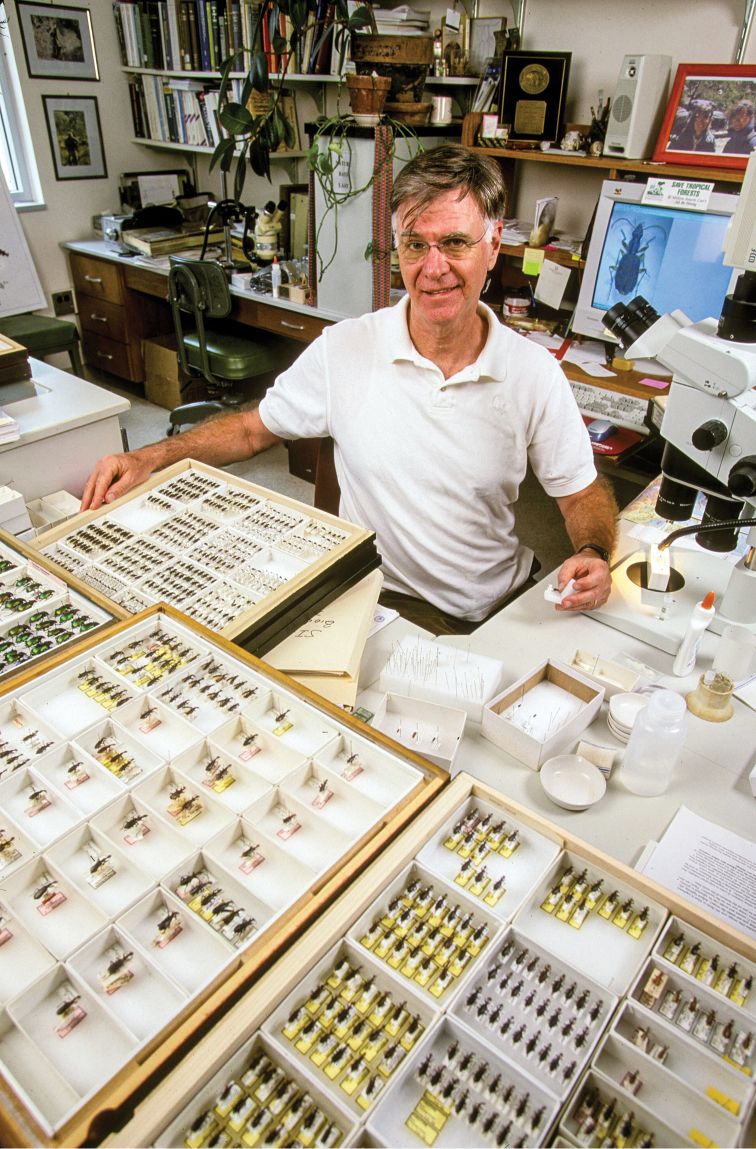
Terry Erwin, Curator of Coleoptera, Smithsonian Institution, 2004.

This interview began in Austin, Texas on 13 November 2013 with The Macallan 18 (a single malt scotch) and a toast “to all things on six legs.” It concluded in Portland, Oregon on 17 November 2014; Erwin was two weeks short of 74 years old.

Rice: What I want to do, Terry, is interview you for a new column in American Entomologist called Legends. And I’ll do this column for five years, or until I run out of energy.

Erwin: [*Laughs.*] This column is a good idea; an excellent idea for ESA.

You are still active in biodiversity and conservation, but I really wanted to narrow this down and look at the entomological aspects and to communicate to entomologists broadly, so some of these questions will be elementary, but some will be philosophical and you just run with it any way you want.

Okay.

Who is the person, or what was the event, that motivated you to study entomology?

Those are always great questions, and I know there are just all kinds of diverse answers you get from everybody, but probably mine is kind of like a common one, and that is J. Gordon Edwards. He was a professor at San Jose State College; it was college then and not university, ’cause this was back in the 60s. My father was a race driver—a tin knocker—and he didn’t finish high school, and when he retired from Mare Island Naval Shipyard, he was a nuclear engineer building atomic submarines, and that was my path. My grandfather worked at Mare Island, my father worked at Mare Island, my mother, my uncle. Vallejo was a very small town, so that was it. You grew up and worked at Mare Island. I actually did four summers there to help pay for my college.

You built atomic submarines to pay for college?

I was working in the atomic reactor room of the Polaris missile [*USS*] *George Washington* submarine. My job was to carry buckets of asbestos mud, so they put it on the preformed stuff, then they would wrap it with a fiberglass cloth. Anyhow, I was waiting for my call. I was just sitting there in the lower level and I leaned up and it was all wet; on my arm was chewing tobacco where somebody above had gone “pitooey.” I looked at that and wiped it off and said this is not for me. [*Laughs.*] So, that was it. I was in junior college taking electives and because I had read a book by James Michener about Hawaii and the Polynesians and their teeth problems, I decided to become a dentist. So I took zoology and thought it was pretty cool. Then I was in a discussion with somebody and they said, “Do you want to spend your life like this?” [*Mimics a dentist staring into a mouth.*]

Leaning over, putting a drill into somebody’s mouth!

Exactly. I just realized I really didn’t want to do that, so I went to San Jose State. I had a favorite English professor in junior college, so I minored in English and majored in life science teaching. During that time, I had to take two life science classes, one of which was marine biology with Polly McMasters and the other was entomology from Gordon Edwards. Polly would get her class up at four o’clock in the morning and go over to Moss Landing and dig up polychaete worms. Frozen fingers and just…gawd! Then we would go back to San Jose and in the afternoon, Gordon Edwards would get out the butterfly nets, and we would go out to Allen Rock Park and collect insects in the warm sunshine. Didn’t take me long to figure out what I wanted to do. [*Laughs.*]

Definitely not a marine biologist?

Definitely not. And also Gordon was just a really dynamic personality, just fantastic. He recruited maybe seven or eight students per year. I just switched to entomology and the interesting thing during that phase was my English classes were dragging me down. I was on probation with a D average and I aced Entomology 51. That was back in the “Pleistocene” when it was 51, not 101. Then I got A’s the entire rest of my student career. It was because of Gordon and his professionalism as a professor and the fact that [when] you brought your insect collection and if there was a *Musca domestica* there, he would just *salivate*, “how great that’s pinned; that’s really a great specimen!” A student just jumps on all that kind of feedback. And that was it.

It is usually one individual and it was Gordon Edwards for you.

He was the one.

Being an “A” student, did you have any challenges during graduate school?

No, actually everybody wanted me to go to Berkeley. There were some great coleopterists there like E. Gorton Linsley. But I said as soon as I walk on campus, I’m going to be the carabid expert, so I’ve got to go somewhere else. I wrote to Carl Lindroth in Sweden, who had just published his volume three of the carabids of Canada and Alaska. So I wrote to him and he said, “Well, you are already working on bombardier beetles, and if you want to do that for your Ph.D., I’m not the right person. You really should go to George Ball.” George who? [*Laughs.*] I wrote George and got back a letter; he was on an 18-month sabbatical in Mexico collecting carabids. He said, “Okay, I’m going to be down here for a little while, but why don’t you just drive on up [to Edmonton, Alberta] and find a place to live and I can support you the first year with pinning my Mexican carabids.” He said just check in with Brian Hocking, the Chair of the department. So my [ex-]wife and I arrived at the Hocking house, and Jocelyn, the wife, opened the door, and she had a black eye and in her arm she held a pet skunk. [*Laughs.*] She was this little British woman with a very nice accent who, unfortunately, connected with a badminton birdie in her eye! “Welcome. They said you were coming.” And so they helped us through the first week and we got a place to live. George supported me for the first year; then I got a Queen Elizabeth Scholarship for the next two years. I finished it in three years.

What was the Queen Elizabeth Scholarship? Was that a full ride?

Yeah, a full-ride scholarship of $2,600 a year. [*Laughs.*] It did fine and that’s actually a Canadian grant. The idea was to finish off as soon as possible. Then Phil Darlington gave me a post-doc and I went from Edmonton to MCZ [Museum of Comparative Zoology]. Then Oscar Cartwright, the old coleopterist at the Smithsonian, retired and they asked George Ball, who was visiting there, “Can you recommend anybody?” He said, “Well, yeah, I just had a student graduate. He’s at Harvard right now, and I’d recommend him.” So they called me and I said, “No, I don’t want to come. I want to do a post-doc with Carl Lindroth in Sweden.” They said, “You can do that too, so come on down.” That’s when I had my first sabbatical. I was in Washington for two months; then I went to Sweden for a year. After that, I had worked with three of the top carabidologists in the world, and that really was my objective.

Let’s jump forward and look at your career. What do you consider your most significant contribution to the field of entomology?

I think this one-page *Coleopterists Bulletin* paper, for one thing, started a cottage industry in fogging, so that became a real technique to look at the forest canopy, and the second thing was a cottage industry in shooting me down [*laughs*] from my naïve hypothesis built on some naïve assumptions, and naïve arithmetic, and coming up with the 30 million [species estimate]. But the point is that most people never realized, that wasn’t the point of the paper. That was a throwaway last paragraph. The point of the paper was that Peter Raven [then Director, Missouri Botanical Garden] called me and he was doing something with the National Research Council, where they needed to know how many species were in an acre of Panama. That was the question. And I said, “Peter, nobody knows that stuff about insects. It’s just impossible.” I had done Panama fogging in the tree *Luhea semannii*, and I said, “Well, give me some time and let me see what I can do.” And so I went through and analyzed all that stuff with those numbers and I came up with 46,000 species per hectare in Panama. He took that and that was great; so let me put this in a little paper for *Coleopterists Bulletin*. Well, if we know this for one tree, how many trees are there in the world? Fifty thousand? Okay, how many insects are host specific? Who knows, but try 13 percent, and so that came to the 30 million. Several people came and said, “Well, what if it’s five percent? What if it’s 20 percent?” And so forth. Those numbers have been batted around and they’re still batted around. The really interesting thing was that Yves Basset, from STRI [Smithsonian Tropical Research Institute], just published a paper last January in *Science*, where he had 110 taxonomists and 10 years of collecting with several different kinds of methods. He came up with a minimum of 28,000 and a maximum of 44,000, based on all of that. And I did it on one tree and some simple math and came up with 46,000! Actually, that’s probably pretty close. We now know that there are probably over 100,000 [species] per hectare in the western Amazon Basin.

**Figure:**
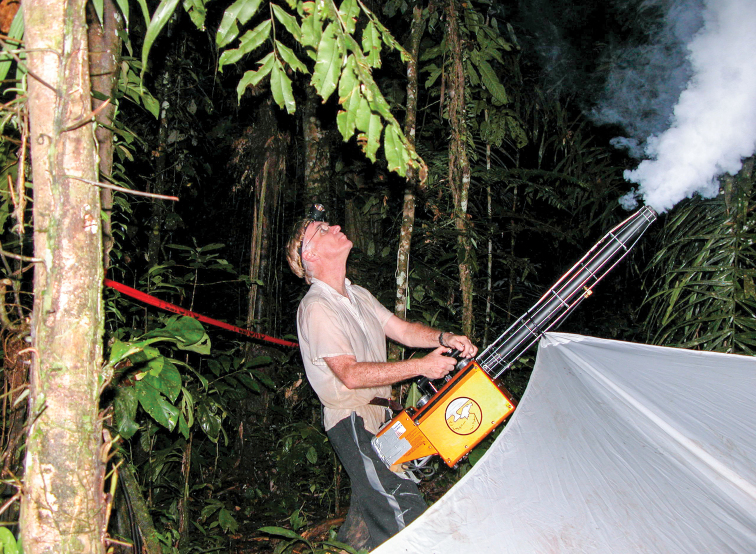
Terry Erwin fogging for insects in the Amazon Basin, 2014.

I checked on the paper in The Coleopterists Bulletin; it has been cited, according to Google Scholar, 835 times.

Yeah, I think it just hit 848^1^.

As long as we are on this number of species, you had estimated 100,000 species per hectare based upon your work in western Ecuador. You have also mentioned 17 billion hectares in the Amazon Basin. Did you provide a number for species?

No. My usual throw-away line is 100,000 species per hectare and 3.210 individuals per hectare in the western Amazon Basin. There are 17 billion hectares and 450 different kinds of forest. Do the math. So that’s my line—do the math.

You are not going to lay a number out there and be quoted?

Right. No. [*Laughs.*] My point this morning [during the symposium] was that the way we collected those things using the garden hose to wash them off the [fogging] sheets, that’s the sample I used to get there. So what if I missed 50 percent of the specimens because they got washed away or…then the 30 million would have been higher.

You have had a tremendous career studying carabids, but why study beetles, and especially beetles that inhabit the rain forest canopy? What got you into the rain forest high up in a tree?

Why don’t we just step back to beetles? Gordon Edwards was the coleopterist and he had a very nice collection and he encouraged us. I started with cerambycids with three of my buddies, who are all cerambycidologists and they were *very* competitive; I mean, *really* competitive. So I asked Gordon if I could have another family. He said, “Well, Carl Lindroth had just published volume three on *Bembidion* and I have been collecting at Glacier National Park and the Tetons and I have lots; you could key those out.” So, that’s how I got into carabids. Before I left for Sweden, Karl Krombein was the Chair [of the department] and he said, “Leave me some proposal about what you are going to do when you come back.” So I wrote a little proposal to do the carabids of California, because I had a hundred thousand [specimens] that I had collected as a student. When I came back, Paul Hurd had taken over as chair and he found out that there was some money to work in Panama, and so he got my proposal—he crossed out California and wrote in Panama. So I ended up, for seven years, working and going back and forth to STRI on Barro Colorado [Island] and that is how I got involved in the fogging. As I said, when those things came down and I saw these rare carabid beetles on the sheet, I said *that’s* how I have got to collect the carabids of the canopy. But it sort of went out of the box from carabids into biodiversity because of the 30-million paper and then that went into conservation and so forth, and the box just kept getting bigger because of that first fogging event.

It was like opening a Christmas package.

Oh man, it’s just unbelievable. And the genus that I’ve been working on for a number of years, which is the genus *Agra* [and] which is strictly canopy—it has turned out to be a lot of fun naming things in the genus *Agra*, but there are just over 500 species described, many of which by me, and in the museum from all my borrows and all my foggings, I have over 2,000 species. So that means I’ve got like 1,600 species that need names. And you know, it’s the last biotic frontier. Until we started fogging, nobody knew what was up there. Now we know the average size of a canopy beetle is 3 millimeters. So you think of architecture: well, the finer you get, the smaller the twigs, the smaller the insects.

Two thousand species of Agra! That has been the focus of your research. I pulled three names off the web: Agra cadabra, Agra vation, and Agra katewinsletae. Give me some context to those names.

*Agra vation* you could guess right away. *Agra cadabra* is a play on words. *Agra dable*—my [current] wife is Peruvian—and this was a very pleasing, nice species. So we speak Spanish and “*agradable*” means very pleasing. [*Laughs.*] *Agra katewinsletae* for Kate Winslet, *Agra liv* for Liv Tyler, *Agra catbellae*, which is Catherine Bell, so all of my heart-beating [*pats his heart and sighs*] female movie stars can get a name *if* they star in a movie where there is a disaster. Okay, so the *Titanic* goes down; in my etymology, the analogy is the destruction of the rainforest and the *Titanic* going down.

Okay.

Liv Tyler was in *Armageddon*, so they are the same thing; the destruction of Earth, the destruction of the rainforest. So all of those celebrity names have to have something to do with disasters. Catherine Bell is a star of *JAG*, a lawyer—she is just luscious. But her nickname was Cat. Did you know Frank Hovore?

Yes, I knew Frank.

Well, Frank used to hit golf balls at the driving range with Cat Bell. He always promised that he would introduce me to her; unfortunately, he died first. But anyhow, he told me her nickname is Cat. So *catbellae* is the species name and I turned that into “the belly of the jaguar” and that was in relationship to the demise of the jaguars’ rainforest.

That’s clever. You have given the names some thought.

Yeah, all of those. We did a Smithsonian Channel hour, two years ago. My post-doc, C. J. Gerasi, and I went into the studio, sort of like a Jay Leno kind of set up, and Susan Spencer is the interviewer. So I come in first and we are chatting a little bit, then C. J. came in and we sat there for a little while and Susan says, “I understand you’ve named some species after movie stars. Did you ever name one after a man?” I said, “Well, of course, after my professor and people who have collected.” “Well, any movie stars?” I said, “Yeah, I did and I had this one species of *Agra* that had this middle femur; big, big femora, so I named it after Arnold Schwarzenegger—*Agra schwarzeneggeri*. That was the one man, and then he became the governor of California.” [*Laughs.*] Then she said, “Oh, yes.” She pulled out this beautiful blue folder with gold lettering from the Office of the Governor of California. My students had done an image of *schwareneggeri* and sent it out to him and he signed it, “Thanks for thinking of me—Arnold.” [*Laughs.*] Anyhow, that is the only one I’ve named after a male star and its physical attributes had nothing to do with movies.

What is your passion in entomology—the thing that most motivates you or brings you the greatest joy?

Curating the national collection. I’m the only coleopterist on the Smithsonian side. I have four USDA colleagues in Coleoptera and each one of them is a contact person for their family. Sasha Konstantinov has chrysomelids, Steve Lingafelter has cerambycids, Lourdes Chamorro is our new curator of weevils, and then Nat Vandenberg is the identifier person. So I have all 165 *other* families in my responsibility and thanks to David Furth, many of those have now been deactivated. So they and my research assistant, Charyn Micheli, and the collection manager for Coleoptera are in charge of 12 million specimens, and of course, nobody can handle that, but then when you bump it down to my responsibility with carabids, we have a little bit over one million carabid beetles. So my goal in my career is to leave that collection just immaculate; as many identified as possible, new species identified as new species, but maybe not described, but everything sorted, everything in perfect order and it is great therapy—just to go in and curate drawers of Coleoptera. Of course, I actually start with the groups I am actively revising and get those done, but then I’m doing a series of books now—the *Carabidae of the Western Hemisphere*. It’s going to be 10 volumes, three are published, the fourth is almost done, I’m starting on five, and there’s 40,000 species of carabids described; just over 10,000 from the Western Hemisphere. So the idea is that’s one legacy project I’m working on, is the ten volumes. To support that is the other legacy, which is to get the collection in perfect shape. That is what I enjoy most.

Describe the experience; when people hear the word Smithsonian, something majestic comes to mind, and for somebody to work there, it’s probably like working in a royal palace.

It is, except the clothes of the royalty are tattered, hand-me-down pants and shoes, [*laughs*] and it’s absolutely awful. My departmental budget, annually for each curator throughout the seven departments, is $2,000. That’s all we get: $2,000. That $2,000 brings me to the ESA meeting every year, and if I want forceps, I have to buy them out of my own pocket. They give us a phone—no charge—and every three years we get an updated computer system—Dell—and updated is not quite correct. What we have to do is take our old ones, turn them back to Dell, then they give us last year’s model. Anyhow, royalty is all a façade, but just the fact is that we have the greatest, accessible collections in the world. Paris [Musée National d’Histoire Naturelle] probably has more specimens, but really not very accessible. You have to go into the attic and look in old boxes and stuff like that. So in that sense, it [the Smithsonian] is a great place to work. The downside is that we can’t go to NSF [National Science Foundation] for funding. I’ve never been able to actually get nice big bunches of money where I could do a five-year project and expect to do it each year, and I just have to beg and borrow year after year after year to do anything. So that part of the Smithsonian really sucks.

Everyone has a story to tell. What is a favorite memory of your career?

In 1976, I think, was the International Congress [of Entomology] in Washington. I decided to do the first international symposium on carabidology and set it up for three days; a symposium with lots of talks. All the carabidologists came; Darlington and Lindroth came over, and I used the State Department to bring some of our folks from behind the Berlin Wall; Fritz Hieke from the Humboldt [University]. In those days, it was really difficult to get those people out [of East Germany]. We had more than a hundred people interested in carabidology and David Maddison was our youngest at 17. Phil Darlington’s talk was about standing on the shoulders of giants. It was just really a dynamic time. I was living in an apartment at that time and Dave Kavanaugh and a couple of my colleagues from Europe were sleeping on the living room floor. You know, it was just really an excellent time. I mean, we’ve had a lot of good times after that, but I think that was a special time.

Back to the Amazon. What do you hope will be the outcome or the long-term impact of your research?

That’s a good question. I’m hoping that as we get the rest of the 2005-2006 and the current samples from this year, get that all in so that we have a 20-year image of what’s going on, then I can tie down these numbers, like the 100,000 species per hectare in *Science* or *Nature* or something like that. That will wake people up to the fact that, yeah, we might have 30 or 50 million species or a lot more on the planet, but we are knocking them off a million at a time. So I think just awareness.

Give me a perspective. When you fog, how much diversity or numbers of things do you find in a year, or how much have you collected in total over all of your efforts over all the years?

Okay. In fogging, the important thing to do is to ask the question and then design the experiment using the fogging system to answer that question. So that may mean you climb a tree and fog just the canopy of that particular tree, or in this recent thing where we are doing bio-monitoring of the oil company road, we wanted actually a picture of the entire forest and see what the impact of the road building and the use of the road by oil trucks is, and so that stretched over twenty years. We just finished up last year; three intense years to start when they were building the road, then the 10-year follow up and the 20-year follow up. We have about nine million specimens from twenty-four hundred samples. Each sample, when you fog standing on the ground up into the canopy, each sample has an average of about 2,800 specimens on a sheet that is three meters by three meters.

Wow! Nine million specimens.

We have 100 sheets for each seasonal visit and we have no idea how many species on that particular sheet, but now as a result of 20 years of looking at everything, doing some extrapolation looking at some taxa, we know how many species there are and the relationship of that taxon with all the published ones. We suspect now that there’s over a hundred thousand species in one hectare of equatorial rainforest in the Western Hemisphere; a hundred thousand species of insects and their relatives and the real Carl Sagan number, the individuals in that hectare, [is] 3.2 times 10 to the 10th individuals. So that figure has no name; you just have to say 10 to the 10th and that’s what we’re getting in a long-term study. If you just go out and fog one tree (one tropical tree, for example), you get about seventeen hundred species; depends on the tree, of course—the next tree might have three thousand species. It just depends on the toxicity of the tree and all kinds of questions like that, but the main thing is, for this oil company road, the rule was that the road could only be 27 meters wide because of various problems Ecuador had with previous oil companies. They put rules and the virgin rainforest had to be intact on both sides of the road, so for insects, after 20 years, there was little or no impact on the entomofauna from that road. However, all the bushmeat was gone in three years. I started with five species of monkeys in the plot; at the end of three years there were no monkeys, no tapirs, no cats, no crassid birds—currasows—anything that was edible was gone.

How far away from the road was this megafauna depleted?

This is the territory of the Huaorani indigenous folks, and before the road, there were 70 dispersed families across two million hectares. [Here’s] a picture of the Huaorani: they have big wooden disks in their earlobes, some of them file their teeth, they don’t have very many clothes, they have blowguns, and they go off for days trying to hit a monkey with a dart. Once the road came in, they dressed in western clothes, they had rifles and they knew how to hitchhike on oil company trucks and this road is 121 kilometers long, so driving back and forth every day hunting, they wiped out the megafauna, or the bushmeat, as we call it, for one to two kilometers back from the road on both sides. The good thing was, 10 years later, most of those old hunters were a little too decrepit to go hunting, and the teenagers—I actually had two teenagers, Huaorani, helping me on the project—they didn’t remember or were never trained on how to follow an old machete trail. So ten years in, my plot had grown in and I asked them to go and clear out this thousand-meter trail to the back of my transect. I was teaching my students how to tie knots and hang up sheets and stuff like that. After half an hour I followed the two [Huaorani] guys and the trail was curving. What’s going on here? I finally caught up with them and they had no idea how to follow scars on the little bushes that were cut ten years before [by machete]. And so, I had two monkey species back in my plot; so there’s hope.

So the monkeys are moving back into the plots.

Yeah.

*I want to take you back to the nine million specimens you collected. What is one of the most unusual things, dramatic things, exciting things that you caught—insect-wise—in your nearly 40 years of fogging in the Amazon*?

One of the very interesting things about these 2,800 specimens that come down, on average per sheet, is once you start parsing out the individuals and looking at the same sheet through the dry season, rainy season, and transition season, which is what we did for each time that we went down, 51 percent of the catch across all 2,400 samples—51 percent were ants. So the majority of abundance is ants no matter where you go. That’s amazing, absolutely amazing. But the next thing that is really, really interesting is you get walking sticks and praying mantids of such camouflage that you just can’t image how these things evolved to blend in with their tree trunks and the leaves and lichens; it’s just amazing. But for me, the most very interesting thing, and I tell this to the hymenopterist at [University of California] Riverside—Heraty, John Heraty. I’ve admitted this to John Heraty and I hate to put it in print, but if the micro-hymenopterists would get off their lazy asses and start describing species, there would be more micro-Hymenoptera than there are Coleoptera.

Really! You think so?

Absolutely, because every beetle, every weevil, has a parasite and those little tiny micro-hym parasites have hyper-parasites of littler micro-hyms. I mean, it’s a no-brainer. But what I wanted to say about that is, when you look under the scope at this tremendous biodiversity that’s in the canopy, the *neatest* thing architecturally are the micro-hyms—they’re just unbelievably fantastic. And don’t tell John, but if I had to do it over again, I might have been a hymenopterist. [*Laughs.*]

*Well, it’s unfortunate that the entomological community can’t see this diversity that you are talking about to learn to appreciate what’s out there*.

That’s the real thing, when you actually get one of these canopy samples and get little spoonfuls in a little plate under the microscope to see the incredible diversity of forms and species and all that kind of stuff that’s in the canopy, that’s actually when you appreciate how much biodiversity’s out there and this hundred thousand species per hectare. Now that’s the Western Hemisphere; the Amazon Basin has 17 billion hectares, and in those 17 billion hectares there’s 450 different types of forest, and each of those forests have *subtypes* of forest within them, so my 30 million estimate is so conservative that it’s just hard to imagine what’s really out there.

So, what’s your new estimate?

It’s impossible to say, absolutely impossible.

I can’t get you to give me a number, can I?

No, no! [*Laughs.*] It’s just impossible to say, but the thing is, I’m getting them on the hoof and we’re looking at morphospecies, but then the gel jocks are going into a species—quote unquote—and finding out that actually that maybe there’s five or six molecular species within that taxonomic species. And so that makes even my samples more diverse than just what you can see with your eyes, and so then, that gives me pause to make another estimate, because they are just getting started with how many siblings are in a morphospecies. So, no—impossible.

*You spent time in the Amazon Basin over a period of several decades. Did you ever encounter a dangerous or threatening situation*?

The first time I was in the Amazon was 1977, so that’s probably 30-plus something years. [*Laughs.*] It seems like longer than that. You know, I’ve seen snakes and all that kind of stuff and been stung by *Paraponera*.

Really? Let’s stop there. Describe being stung by the bullet ant.

It’s a *real* shock when you get stung and you know *immediately* what it was. The first time was on the back of my arm.

You’ve been stung more than once?

Yeah. So I grabbed that thing and pulled it out, and I forget who was with me, and they looked all over and there was another one on my leg and they flipped that off, so I didn’t have any problems. I was just squeezing and squeezing, and then it dropped out, but they are so hard I didn’t kill it and it was crawling away. That lasted for about half an hour, and by day two, oh, then after the fire, it goes to a feeling like a dull toothache and the toothache kind of goes for a couple of days and then it’s gone.

You mentioned a fire. Do you mean that the sting felt like you had been burned?

Right. It’s a severe burning sensation. The second time was in this oil company transect on the road and the oil company film team had come out and they were doing interviews in the forest and they wanted me to stand over there. I was just standing and not paying attention, but [I] was next to a *Paraponera* nest at the base of a tree and one crawled up, went out on my arm while I am giving the interview, and it stung me in the thumb. Being stung on any of the fingers is the worst thing possible.

Because why?

I think we have more nerve endings in our fingertips. It’s a nerve agent, what they are actually putting in there. That film has more four-letter words than I [*laughs*] probably even I know in my conscious. I jumped up and ran in circles and they were filming me and wondering what the hell I’m doing, and I’m cussing and swearing and shaking my hand. Of course I knew exactly what it was because I had a previous experience. So those are two of my *Paraponera* experiences.

What is the most dangerous thing in the rain forest?

The most dangerous thing is actually a tree fall, or a branch fall. A good-sized branch comes down pretty fast and if you’re under it and get hit on the head—you’re dead. That’s it. A tree fall, it takes a while, and you hear the crack, you look and if it’s coming toward you, you just step one meter [aside] and it misses you. If you *run*, you don’t know where it is coming down and it could just clobber you. It’s the branches that come down that are more dangerous. One time in Tambopata [Peru] I was taking down my pulleys for pulling up the fogger, and I was pulling it out and a branch broke about that big [*makes a circle with his hands the size of a baseball*], and when they break, they kind of have a pointy thing on them and it came down and went through the hair here [*points to his forehead*], didn’t hit my nose, but the branch went down, ripped my chest clothes a little bit and then ripped the material in my crotch and stuck in the ground between my legs. It was a long branch, so I’m sort of looking through the foliage and all my colleagues are standing around kind of laughing a little bit until they actually realize what happened. If it had hit me in the head, I’d be dead. When I got back [to camp], I noticed my underwear also was ripped right out, but nothing on my body. No scratches. It was so close it just took out my clothes. So that is the only time in 40 years that I have been doing fieldwork in the rain forest that anything *close* to being a disaster happened.

Do you have a favorite insect species? It has to be a carabid.

Oh, absolutely. My license plate says AGRA DAX, and Dax is from *Star Trek: Deep Space Nine*, and the actress, Terry Farrell—beautiful woman, absolutely beautiful. She played Dax, and actually it was Terry Farrell’s body, but Dax is actually an alien parasite that lives in her, but the alien was so ugly that it had to have a different body, and what a body! Anyhow, *Agra dax* is my favorite. It’s actually a very large *Agra* with a heavy body from Panama and metallic green with a rufous head with black antennal segments, so it’s quite colorful, and this particular group has flattened tibiae and femorae, which means it probably lives with ants, but we don’t know too much about it. Its sister species are *Agra sasquatch* and *Agra yeti*. Why? Because they have these *really* expanded tarsal segments, so then it’s like Bigfoot.

**Figure:**
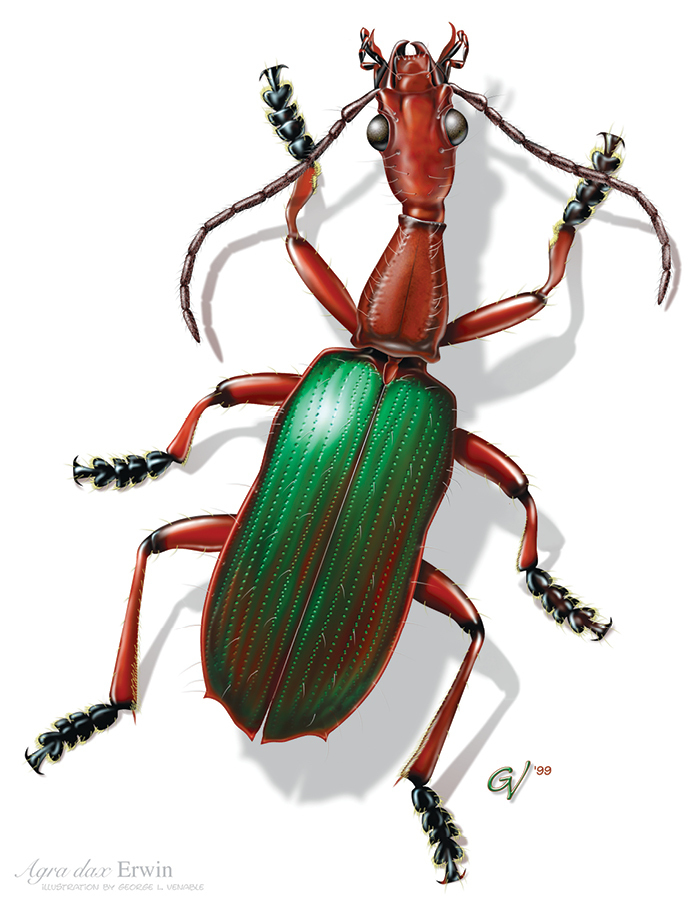
*Agra dax*.

**Figure:**
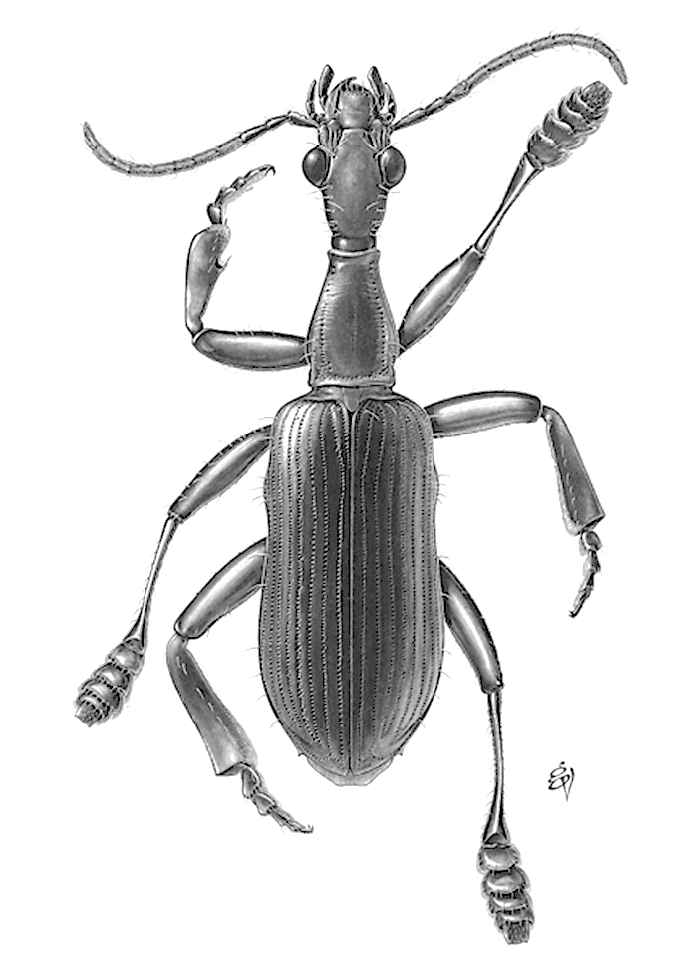
*Agra sasquatch*.

What do you consider to be your legacy, or how do you want others to remember you?

I guess maybe by what my students do. If I’ve influenced my students in a good way and they go on to do stuff, then the unbroken chain just keeps going. So that’s George Ball; he had 40 Ph.D. students, not just in carabids, but in other taxa, as well, and many of us have gone on like Dave Kavanaugh—my best friend and [previous] Chair and Science Director at the California Academy of Sciences. So you go back to George and to Cornell, you have Forbes, and you go back from him to Cuvier and Buffon, so there’s this chain all the way from the great old-timers down through George and his students and what I’d like to do is to keep that chain going with my current student, Laura Zamorano from Colombia, and others.

I hope I have that much energy when I reach your age.

I’ve now lived in the Amazon for 16 years of my life with the various expeditions all put together, so for 16 years I breathed absolutely pure oxygen. [*Laughs.*] So that’s a plus. And beetles are my hobby, as well as profession. I *never* have any stress. If there’s something not quite going right, I go curate a drawer of beetles, you know. My current wife, Grace Servat, is Peruvian and is quite a bit younger than me and she kicks my ass if I’m lying around, or something. [*Laughs.*] She’s an avian ecologist that specializes in the high Andes. So she’s up at 4,500 to 5,000 meters in her cushion-plant zone at the very top breathing more than pure oxygen. I’ve been with her a couple of times when there is no oxygen for my lowland Amazon lungs, so now I just have her show me pictures and tell me about it. She does the same for my lowlands; she hates it down where there’s biting bugs and [it’s] hot and sweaty. So we do our own research, then come back to talk about it, which is exciting to hear.

What is the compromise?

The compromise is our house in Washington; we come back to the home base and our garden.

Terry, I greatly appreciate your candidness in answering my questions.

Well, it was fun, and The Macallan 18 single malt scotch helped, too!

Whenever I see you at an ESA meeting, you always have a cloud of people hovering around you.

Most of them are students; younger people. The students keep you young. Like I said, all my students want me for another 30 years. “You can’t go!” [they say]. And I’m not!

**Figure:**
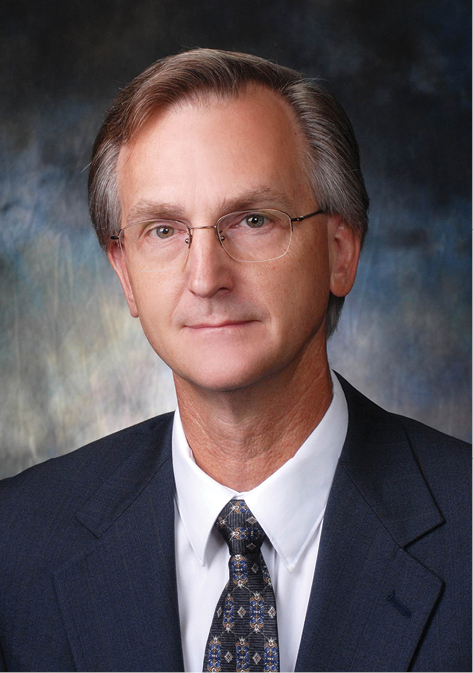
Marlin E Rice is a research manager with DuPont Pioneer in Johnston, Iowa. He is a past President and Fellow of the Entomological Society of America.

## Footnotes

920 citations as of 1 January 2015.
